# Integration of resting state functional MRI into clinical practice - A large single institution experience

**DOI:** 10.1371/journal.pone.0198349

**Published:** 2018-06-22

**Authors:** Eric C. Leuthardt, Gloria Guzman, S. Kathleen Bandt, Carl Hacker, Ananth K. Vellimana, David Limbrick, Mikhail Milchenko, Pamela Lamontagne, Benjamin Speidel, Jarod Roland, Michelle Miller-Thomas, Abraham Z. Snyder, Daniel Marcus, Joshua Shimony, Tammie L. S. Benzinger

**Affiliations:** 1 Department of Neurological Surgery, Washington University School of Medicine, St. Louis, MO, United States of America; 2 Center for Innovation in Neuroscience and Technology, Washington University School of Medicine, St. Louis, MO, United States of America; 3 Brain Laser Center, Washington University School of Medicine, St. Louis, MO, United States of America; 4 Department of Biomedical Engineering, Washington University in St. Louis, St. Louis, MO, United States of America; 5 Department of Mechanical Engineering and Material Sciences, Washington University in St. Louis, St. Louis, MO, United States of America; 6 Mallinckrodt Institute of Radiology, Washington University, St. Louis, MO United States of America; Banner Alzheimer's Institute, UNITED STATES

## Abstract

Functional magnetic resonance imaging (fMRI) is an important tool for pre-surgical evaluation of eloquent cortex. Classic task-based paradigms require patient participation and individual imaging sequence acquisitions for each functional domain that is being assessed. Resting state fMRI (rs-fMRI), however, enables functional localization without patient participation and can evaluate numerous functional domains with a single imaging session. To date, post-processing of this resting state data has been resource intensive, which limits its widespread application for routine clinical use. Through a novel automated algorithm and advanced imaging IT structure, we report the clinical application and the large-scale integration of rs-fMRI into routine neurosurgical practice. One hundred and ninety one consecutive patients underwent a 3T rs-fMRI, 83 of whom also underwent both motor and language task-based fMRI. Data were processed using a novel, automated, multi-layer perceptron algorithm and integrated into stereotactic navigation using a streamlined IT imaging pipeline. One hundred eighty-five studies were performed for intracranial neoplasm, 14 for refractory epilepsy and 33 for vascular malformations or other neurological disorders. Failure rate of rs-fMRI of 13% was significantly better than that for task-based fMRI (38.5%,) (p <0.001). In conclusion, at Washington University in St. Louis, rs-fMRI has become an integral part of standard imaging for neurosurgical planning. Resting state fMRI can be used in all patients, and due to its lower failure rate than task-based fMRI, it is useful for patients who are unable to cooperate with task-based studies.

## Introduction

Striking the correct balance between aggressive resection and functional preservation has been an ongoing dynamic in neurosurgery. Metrics that predict long-term survival of malignant glial neoplasm are 1) extent of resection, and 2) preservation of functional status[1–3]. Functional preservation historically has relied on the gold standard of intraoperative cortical stimulation[4]. More recently, task-based functional magnetic resonance imaging (fMRI) has also become a useful adjunct for pre-surgical planning[5]. Task-based fMRI can be associated with a high failure rate in populations that cannot comply with task paradigms (e.g., cognitively/neurologically impaired patients, and children). Moreover, each function must be individually mapped, making the mapping paradigms lengthy. These limitations are eliminated by the use of resting state functional magnetic resonance imaging (rsfMRI). This approach uses the endogenous brain activity detectable with blood oxygen level dependent (BOLD) MRI to identify areas that are interacting at rest[6]. Spontaneous BOLD fluctuations are low-frequency (<0.1 Hz) oscillations in metabolic activity that are anatomically correlated within distinct functional networks[7]. Using spontaneous activity, one can generate resting-state correlation maps that are similar to the functional maps obtained from task activations[8]. rsfMRI is highly efficient: multiple resting state networks (RSNs) (e.g. motor, speech, etc), can be mapped simultaneously with a single imaging session lasting less than fifteen minutes[9]. The imaging can also be performed under sedation/anesthesia without compromise of the functional localization[10].

The current barrier to widespread use of rsfMRI in neurosurgical practice is the high degree of advanced imaging expertise necessary to perform the analyses. To address this shortcoming we have done two things. First, we have created a multi-layer perceptron (MLP)-based analysis tool that assigns RSN membership (e.g., somatomotor, language, etc) to each locus within the brain. We have shown that MLP-based RSN mapping is a powerful tool for automating the identification of resting state networks in individuals[9]. MLP-based RSN mapping has been shown to be useful in presurgical planning and shown to correspond to results obtained by cortical stimulation[11]. Second, we have created a dedicated imaging informatics platform that seamlessly integrates MLP-based RSN mapping into the clinical IT infrastructure[12]. The RSN mapping system has been configured such that all algorithms/calculations can be performed rapidly on a single computer and interface with clinical Picture Archiving and Communication System (PACS) systems and the Medtronic StealthStation Navigation System.

In this study we retrospectively report on the initial eighteen-month experience of clinical deployment of this integrated imaging platform. Cumulatively, the experience demonstrates robust neurosurgeon adoption, utilization for a diversity of clinical applications, and a reduced failure rate when compared to standard task-based functional mapping.

## Methods

### Patient characteristics

After obtaining institutional review board approval from the Washington University Human Research Protection Office, the MRI of 191 consecutive patients between January 1, 2014 to June 30^th^, 2015 who had a rs-fMRI underwent retrospective evaluation of the images and electronic medical record for data collection. The patient demographics and underlying disease that were evaluated with rs-fMRI are summarized in Table 1.

**Table 1:** Patient demographics.

**Patient Demographics**	
**Total patients**	N = 191
**Male**	N = 118 (62%)
**Female**	N = 73 (38%)
**Age**	46 y/o (3–84 y/o)
**Adult**	N = 173 (91%)
**Children**	N = 18 (9%)
**Tumor**	Low grade glioma N = 41 (21%) High grade glioma N = 80 (42%) Other tumors (DNET, Meningioma, Pilocytic Astrocytoma, Ganglioglioma) N = 32 (17%)
**Vascular Malformations**	N = 10 (5%)
**Epilepsy**	N = 6 (3%)
**Non-neoplastic (Encephalitis, OCD** **Neurocysticercosis, Amyloid angiopathy, CJD)**	N = 12 (6%)
**Negative pathology**	N = 6 (3%)
**Pathology N/A (4/191)**	N = 4 (2%)

### Pre-processing pipeline

The pre-processing used in the present work has been described elsewhere[13]. Briefly, this includes compensation for slice-dependent time shifts, elimination of systematic odd-even slice intensity differences due to interleaved acquisition and rigid body correction for head movement within and across runs. The fMRI data are intensity scaled (one multiplicative factor applied to all voxels of all frames within each run) to obtain a mode value of 1000. Atlas transformation is achieved by composition of affine transforms connecting the fMRI volumes with the T1- and T2-weighed structural images. Head movement correction is included in a single resampling to generate a volumetric time-series in 3mm^3^ atlas space.

Additional preprocessing in preparation for MLP analysis includes the following: (1) spatial smoothing (6 mm full-width half- maximum Gaussian blur in each direction), (2) voxelwise removal of linear trends over each run, (3) temporal low-pass filtering to retain frequencies < 0.1 Hz, and (4) reduction of spurious variance by regression of nuisance waveforms derived from head motion correction and extraction of the time series from regions of noninterest in white matter and CSF. This step includes regression of the global signal averaged over the whole brain. Global signal has demonstrated enhancement in the detection of system-specific correlations [14]and exhibits shared variance with respiratory and cardiac signal regressors[15]. The sensitivity of the fMRI signal to arterial pCO2 fluctuations and head motion is well described, Our standard pre-processing procedures include (5) scrubbing techniques to remove volumes contaminated by large head movements[16]. Systemic evaluation of the most common pre-processing techniques have demonstrated that our methods are representative of the most effective artifact reduction strategies[17].

### The multi-layer perceptron

The topography of resting state networks (RSNs) in individual patients was mapped using a multilayer perceptron (MLP) trained to assign RSN membership to each voxel on the basis of resting state fMRI (rs-fMRI) data. A full technical description of this method is given in [9]. The following summarizes the essential points. MLPs are supervised classifiers trained to map input data, i.e., functional connectivity maps, to pre-defined output classes, e.g., RSNs. The critical distinction between MLP-based RSN mapping vs. alternative strategies, e.g., ICA [18] is that the former is supervised while the latter is not. To illustrate by analogy, automatic number readers are supervised classifiers, used by the post office to automatically route letters according to zip code[19]. Such classifiers are trained by presentation of hand-written numbers together with their associated true identity. An unsupervised classifier would have to first deduce, by exposure to hand-written zip-codes, the existence of a set of archetypical number symbols (e.g., "2", "8", etc.). It is obvious that unsupervised classification is a vastly more difficult challenge in comparison to supervised classification.

The MLP was trained to associate correlation maps with a priori defined, resting state network identities. The seven RSN were as follows: default mode network (DMN)[20, 21], sensorimotor network (SMN)[7], visual network (VIS)[18, 22], language network (LAN)[18, 22], dorsal and ventral attention network (DAN, VAN)[23, 24], and the fronto-parietal control network (FPC). RSNs were defined using task-fMRI responses in 21 normal young adults screened to exclude neurological impairment and psychotropic medications[9]. Importantly, prior to initial MLP training, this ROI set was iteratively refined to ensure consistent (across subjects) correlation maps across all seed ROIs assigned to a given RSN. This preliminary refinement step generated 169 canonical seed ROIs representing 7 RSNs. Following initial training, the MLP architecture was optimized using a separate dataset (N = 17). Performance of the trained MLP then was validated in separate, very large (N = 692) dataset obtained from the Harvard-MGH Brain Genomics Superstruct Project[25]. This validation demonstrated reliable classification RSN membership of cerebellar voxels[26], even though the cerebellum was excluded from the training dataset. It was also shown, using a second validation dataset (N = 10), that the somatomotor RSN was reliably mapped over the central sulcus despite cm-scale, intra-individual differences in gyral anatomy.

### Imaging workflow

Standardized order sets for resting-state fMRI with or without a complete conventional brain MRI or task-based fMRI were created to facilitate clear communication of orders between the neurosurgeons and the radiology department. Examinations are scheduled for the first available time slot and entered into a tracking calendar to allow technical and professional staff to track the case to completion.

All patients were scanned using a 3-T TRIO scanner (Siemens, Erlangen, Germany). rs-fMRI data were acquired using a T2* EPI sequence (3 × 3 × 3-mm^3^ voxels; 128 volumes/run; TE = 27 ms; TR = 2.8 s; field of view = 256 mm; flip angle = 90°), while the patients were instructed to remain still and fixate on a visual cross-hair without falling asleep (2 runs of 6 minute each for a total time of 12 minutes). Tumor protocol anatomic imaging included T1-weighted magnetization-prepared rapid acquisition gradient echo (MP-PAGE), T2-weighted fast spin echo, susceptibility-weighted imaging (SWI), diffusion-weighted imaging (DWI) and pre and post gadolinium T1-weigted fast spin echo in multiple projections. All anatomic and functional magnetic resonance data were acquired in approximately 60 minutes for each patient.

Our workflow was streamlined to facilitate acquisition of the rs-fMRI data and rapidly transfer data before and after processing using a system referred to as the Translational Imaging Portal (TIP). TIP is a customized version of the XNAT imaging informatics platform[12] that was created to facilitate the translation of imaging research into clinical practice. XNAT is a web-based software platform designed to facilitate common management and productivity tasks for imaging and associated data. It consists of an image repository to store raw and post-processed images, a database to store metadata and non-imaging measures, and user interface tools for accessing, querying, visualizing, and exploring data. XNAT supports all common imaging methods, and its data model can be extended to capture virtually any related metadata. XNAT includes a DICOM workflow to enable exams to be sent directly from scanners, PACS, and other DICOM devices. XNAT’s web application provides a number of productivity features, including data entry forms, searching, reports of experimental data, upload/download tools, access to standard laboratory processing pipelines, and an online image viewer. A fine- grained access control system ensures that users are restricted to accessing only authorized data. XNAT also includes a web services API for programmatic access and an open plugin architecture for extending XNAT’s core capabilities.

While the standard open source XNAT platform provides the infrastructure for securely storing, processing, and reviewing the rs-fMRI studies, TIP extends XNAT in several ways to support the full clinical rs-fMRI workflow. An interface was implemented to query and retrieve patient MRI studies from the clinical image archive. The interface allows patient studies to be easily reviewed to identify those containing resting state sequences. Fully automated pipelines were implemented to orchestrate the actual execution of the perceptron and perceptron quality control algorithms. These pipelines ensure that the algorithms are executed consistently and that all parameters and execution history is fully documented. The pipelines execute the algorithms and generate individual DICOM-formatted images representing each resting state network. The MLP output provides a probability for the classification of each voxel to a certain network. The network maps were provided to the surgeons at several probability thresholds, allowing the surgeon to select the one that makes the best representation for the surgery. The most popular choice has been the 97% threshold. These images are posted to the XNAT database and integrated with the original imaging exam. Finally, custom screens were added to the XNAT web-based user interface to provide patient-centric views of the cases and to implement a stepwise workflow for processing and reviewing cases. This stepwise procedure enforces a standardized procedure that includes careful review of processed results before the network maps can be submitted to the clinical archive. TIP includes reporting features to track performance and quality metrics over time.

Technical staff trained in running the resting-state fMRI processing pipeline retrieve the case from PACS, process the case, review the quality control parameters, and upload the processed resting-state fMRI images back to PACS and the neurosurgical intraoperative planning workstation for integration with diffusion tensor tractography.

**Fig 1:**
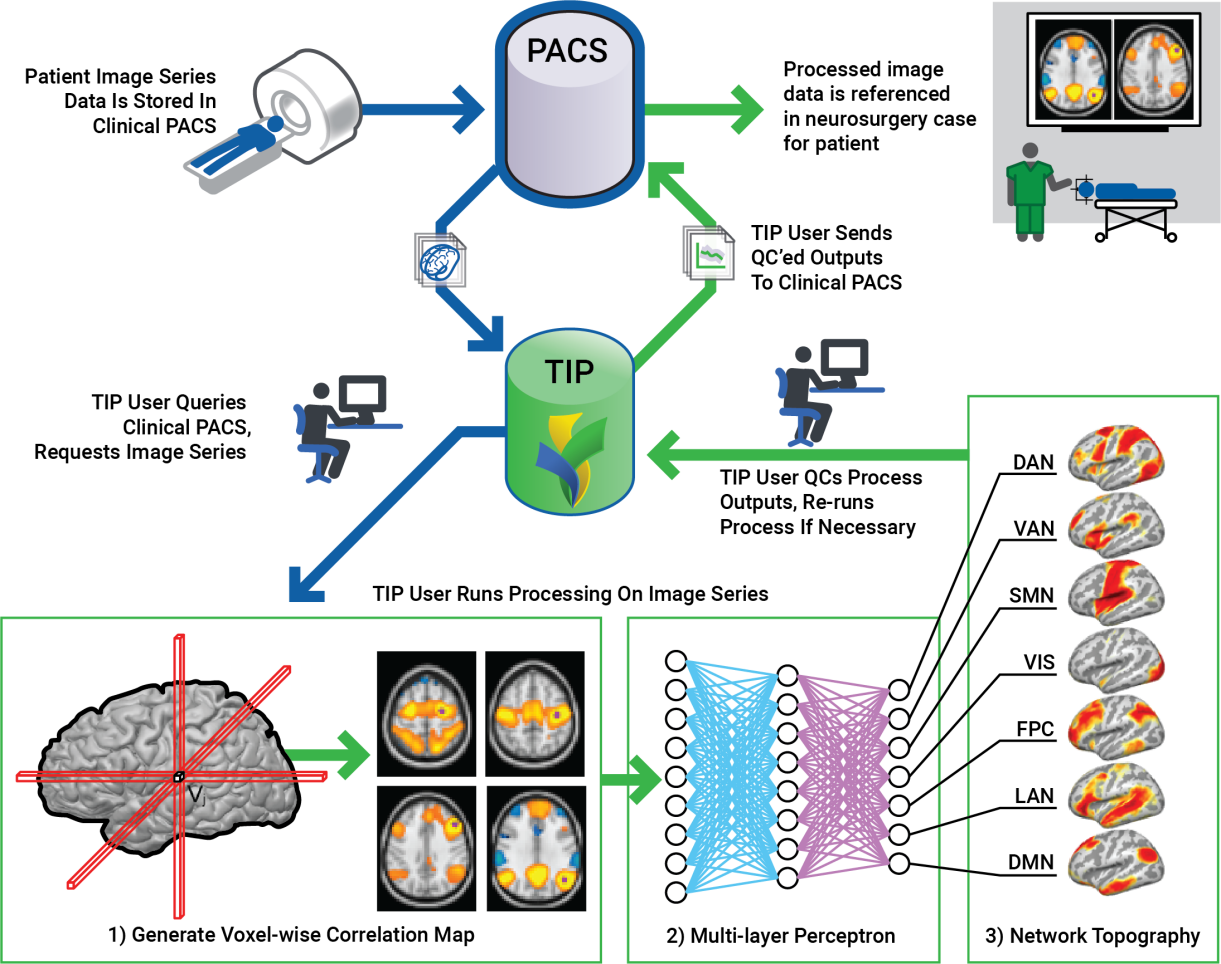
Resting state processing workflow. The Translational Imaging Portal (TIP) provides informatics capabilities to (1) retrieve MRI exams from the hospital clinical information system (PACS), (2) run the automated rsfMRI processing pipeline via the multi-layer perceptron analytic on a HIPAA-compliant computing cluster, (3) review quality control metrics generated by the pipeline, and (4) submit MLP maps for exams that pass QC to the PACS.

**Fig 2:**
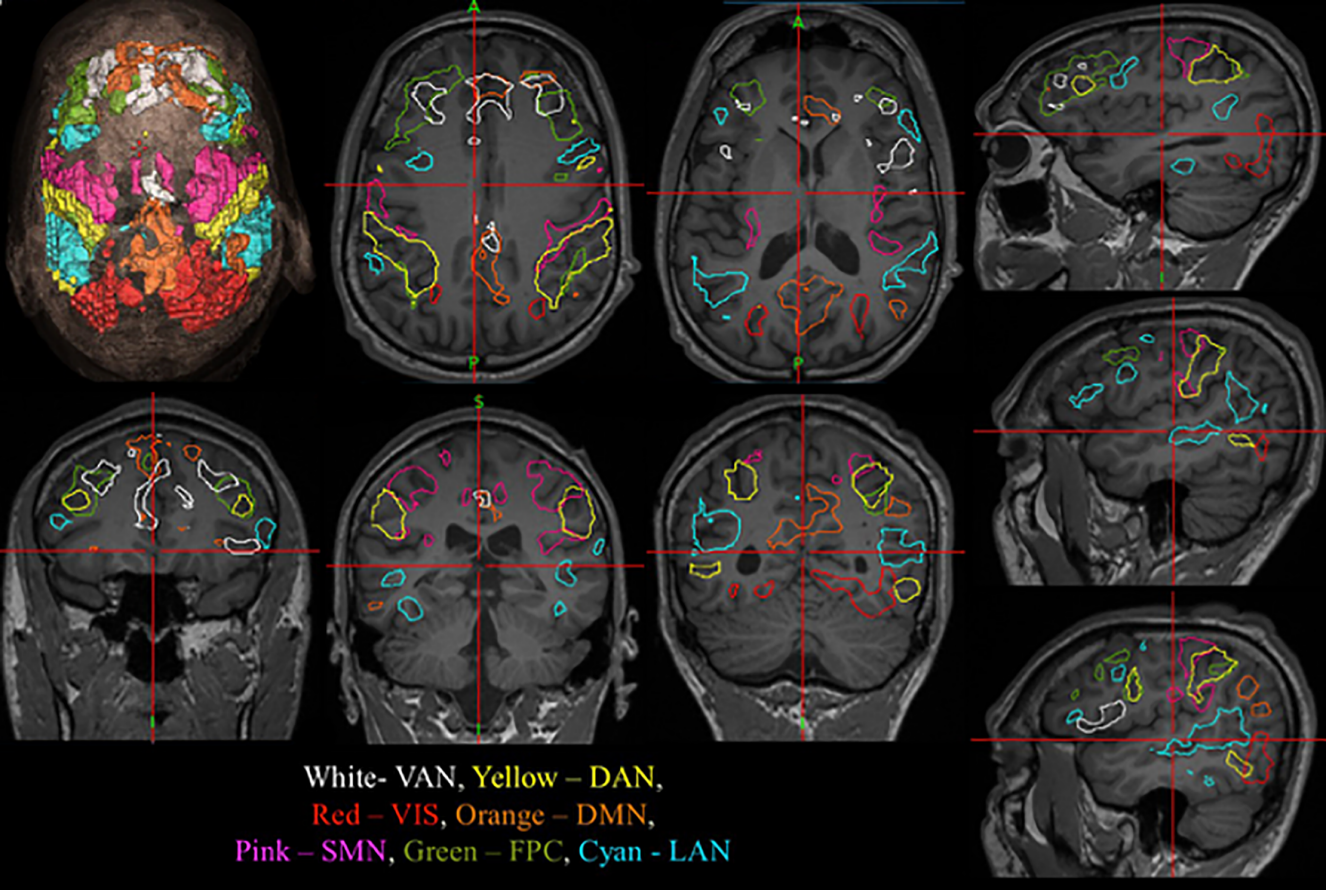
Resting state networks shown in stealth navigation software. After acquisition of rs-fMRI and processing of the data through the MLP analytic, the images are uploaded to the Medtronic Stealth Station. The workflow was streamlined to facilitate acquisition of the rs-fMRI data and rapidly transfer data before and after processing using a system referred to as the Translational Imaging Portal (TIP). The seven RSN were as follows: Default Mode network (DMN), Sensorimotor network (SMN), Visual network (VIS), Language network (LAN), Dorsal and Ventral Attention network (DAN, VAN), Fronto-Parietal Control network (FPC).

Quality control measures include assessment of translational and rotational motion, and co-registration of the BOLD data with the anatomical image. The time required to process clinical resting-state fMRI in the processing pipeline varies from thirty minutes to one hour depending on whether or not the case passes the quality control steps without modification. The interpreting radiologist reviews and reports on the resting-state fMRI, conventional MR and task-based fMRI if available, and diffusion tensor tractography. The scan is then uploaded to the intraoperative navigation system in preparation for the surgery. A schematic of the workflow pipeline from image acquisition to surgical navigation is provided in Fig 1. An example of imaging of all resting state networks downloaded to the intraoperative navigation system is shown in Fig 2.

### Post-processed imaging data evaluation

Patients were identified who had concurrent task-based fMRI (tb-MRI) and resting state fMRI. All studies were evaluated as successful or failed with regard to demonstrating clinically relevant topographies. Specifically, we compared in the subset of eighty-three patients that received both resting state fMRI and task-based fMRI the failure rate in which no functional localization was accomplished.

## Results

### Clinical utilization and alteration of practice patterns

A total of 191 consecutive patients (173 adults and 18 children) underwent a total of 232 rs-fMRI sessions between January 1, 2014 and June 30, 2015. One hundred fifty-five patients had a single rs-fMRI session, 31 patients had 2 rs-fMRI sessions and 5 patients had 3 rs-fMRI sessions. Overall utilization increased between January 2014 and June 2015. One hundred eighty-five studies were performed in the setting of intracranial neoplasm, either primary or metastatic, 14 studies were performed in patients with epilepsy and 33 studies were performed in the setting of other neurologic disorders (including vascular malformations, inflammatory or infectious disorders, as well as neuropsychiatric disorders). See Table 1 for additional demographic information. For the neurosurgical patients, 76% were used in the context of a craniotomy, 15% with laser interstitial therapy, and 9% with biopsy.

### Study of failure rates

Of the 191 unique patients undergoing rs-fMRI, 83 also underwent both motor and language task-based fMRI. Thirty-two task-based studies failed (38.5%, 32/83) while 28 rs-fMRI sessions failed (13%, 28/232). These differences were statistically significant p < .0001 (Fischer’s exact test) with rs-fMRI having a significantly reduced rate of imaging failure. Causes of failure for both study types included lack of cortical activation despite appropriate thresholding, motion or susceptibility artifact, lack of cooperation, lack of MPRAGE image acquisition for registration, registration errors and technical errors such as incorrect TE/TR parameters (see Table 2).

**Table 2:** Comparison of performance between task-fMRI and resting state fMRI.

**A. Cause Of Failure**	rs-fMRI (n = 232)	task-based fMRI (n = 83)
Patient Motion	14 (6.0%)	0 (0%)
Susceptibility artifact	6 (2.6%)	0 (0%)
Unable to follow commands	0 (0%)	10 (12%)
No activation	2 (.9%)	20 (24%)
Technical failure NOS / did not pass QA	3 (1.2%)	2 (2.5%)
Anatomic misregistration to atlas	3 (1.2%)	0 (0%)
**Total**	**28 (13%)**	**32 (38.5%)**
**B. Performance Between rs-fMFI and task-fMRI**		
	**Successful rs-fMRI**	**Failed task-fMRI**
**Successful task-fMRI**	**54**	**25**
**Failed task-fMRI**	**29**	**3**

Most resting state failures were due to motion that occurs during the resting state acquisition when the patients fall asleep, have involuntary movements, or forget instructions to hold still. This typically does not occur with the successful task-fMRI because the patient can remain still and awake while concentrating on a task to perform. The anatomic distortion is due to extreme cases of mass effect or large territories of prior resection. The resting state processing algorithm generally handled moderately large tumors or regions of resected brain quite well. The signal loss due to susceptibility occurred in one pediatric patient with braces and in a few patients who recently had resections or had recent hemorrhage causing signal loss next to large collections of blood or postoperative gas.

### Exemplar cases

Here some exemplar cases are presented that demonstrate the fundamental utility of rsfMRI.

#### 1. Comparable localization between task and resting-state fMRI

**Fig 3:**
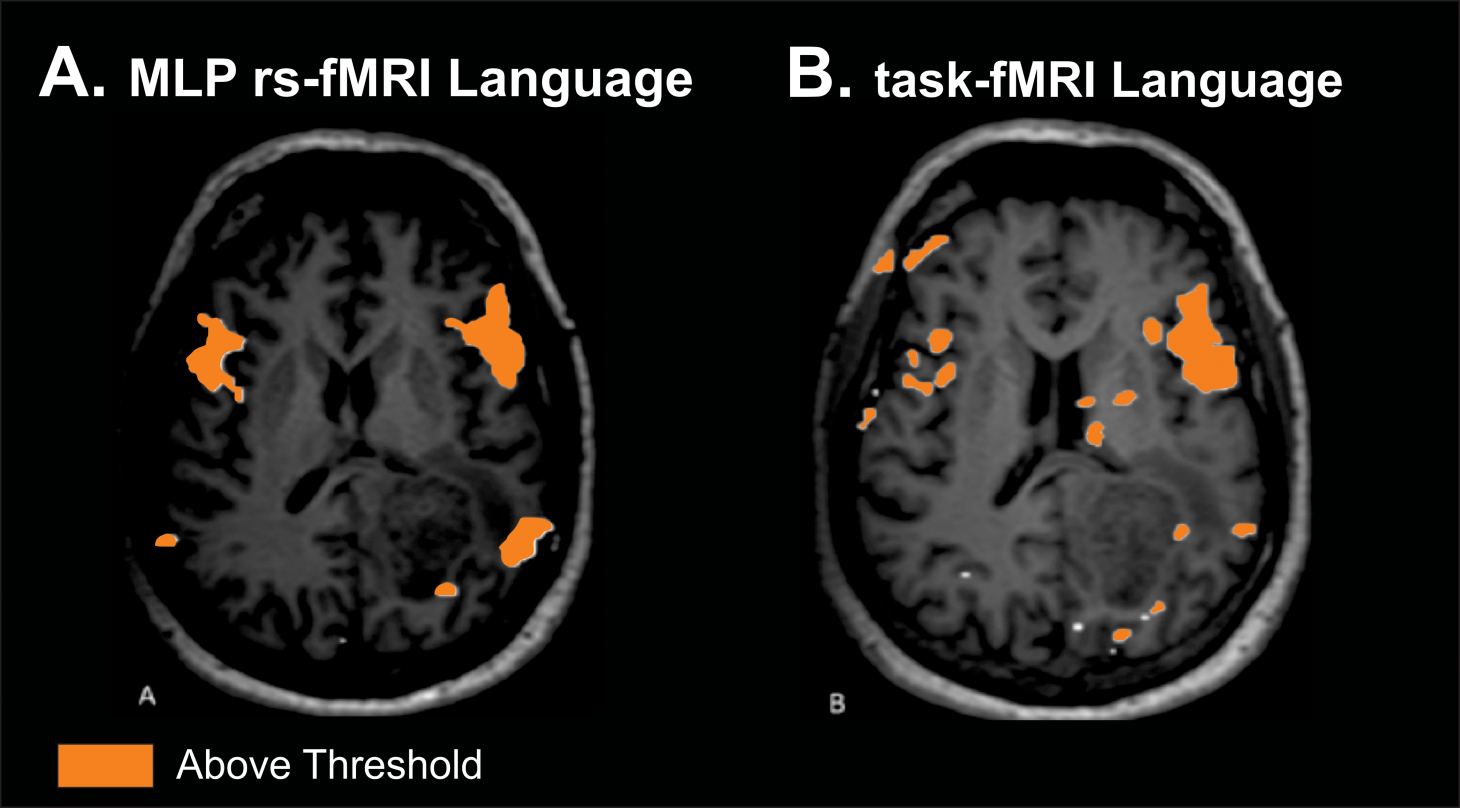
Comparable localization between task and resting-state fMRI. Single patient comparison for language for both MLP rsfMRI (A) and task (B). Both demonstrate similar regions of topographic localization of function. Imaging threshold for MLP rsfMRI was 97% probability. Threshold for task was above threshold as defined by clinical imaging software package.

**Fig 4:**
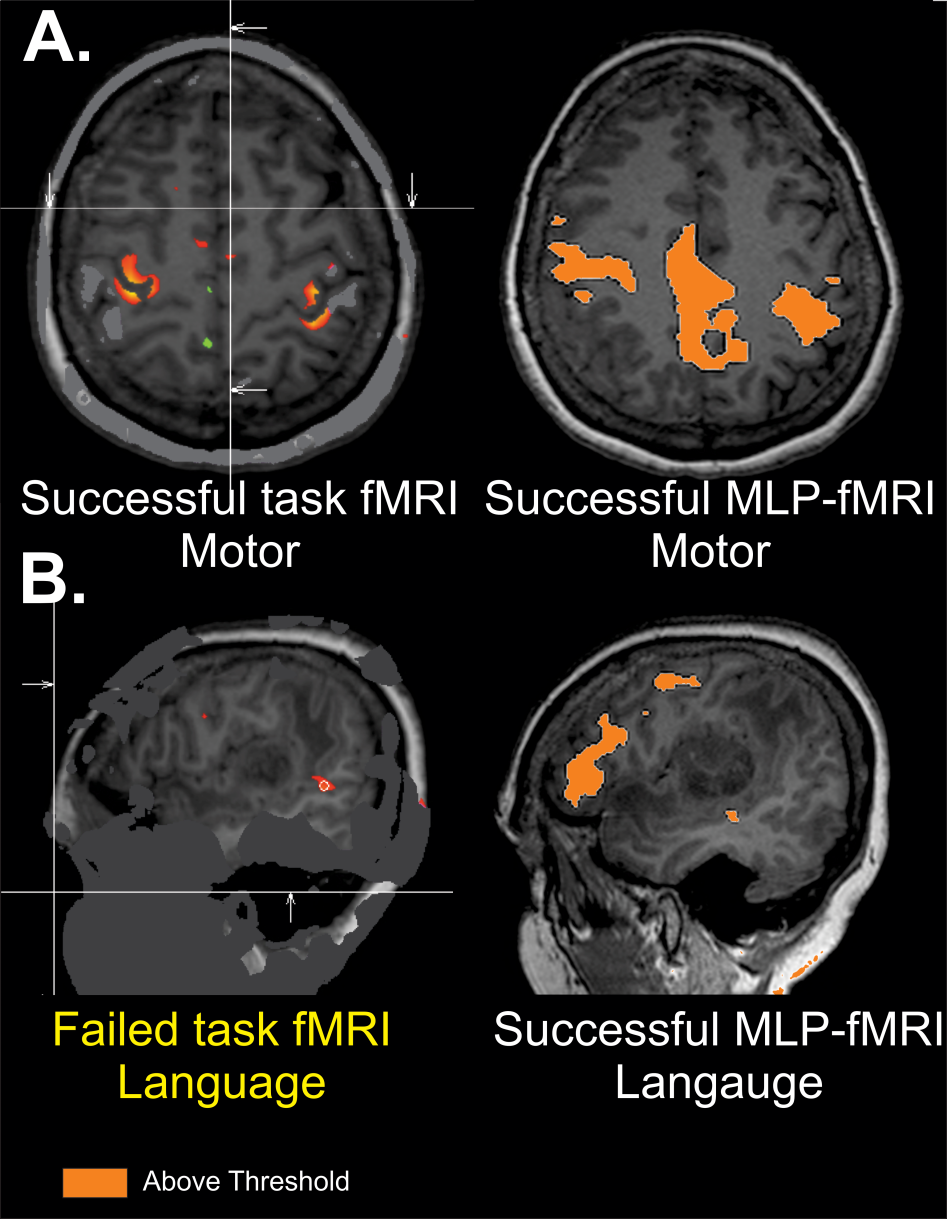
Successful rsfMRI mapping in the setting of failed task-based fMRI. Single patient comparison for motor and language for both task and MLP rsfMRI. While both modalities are successful for motor which is distant from the tumor (A), task-based imaging does not show localization in the frontal lobe, while MLP-resting state fMRI does show good localization (B). Imaging threshold for MLP rsfMRI was 97% probability. Threshold for task was above threshold as defined by clinical imaging software package.

Patient is a sixty-two year-old with left parietal biopsy-proven glioblastoma multiforme. Resting state fMRI is shown in Fig 3A and task-based fMRI is shown in Fig 4B Broca’s area activation bilaterally and Wernicke area activation on the left are similar for both techniques.

#### 2. Successful rsfMRI mapping in the setting of failed task-based fMRI

Patient is a fifty-year-old male with new onset of headache. Preoperative functional imaging demonstrates a large mass in the posterior inferior frontal lobe. Fig 4A shows motor system results with MLP-rsfMRI and for task-based fMRI (1&2). Both show reasonable functional localization around the central sulcus. In the same patient, Fig 4B shows language system results for MLP-rsfMRI and for task fMRI. While there is no evidence of cortical activation with in the frontal lobe with task-based fMRI for language (4B), there is a strong functionally localized region with MLP-defined resting-state MRI (4B). Taken together, these findings show MLP-resting state fMRI has the capability of identifying functional cortex in tenuous regions where task-based fMRI fails.

#### 3. Mapping speech sites in an aphasic patient

**Fig 5:**
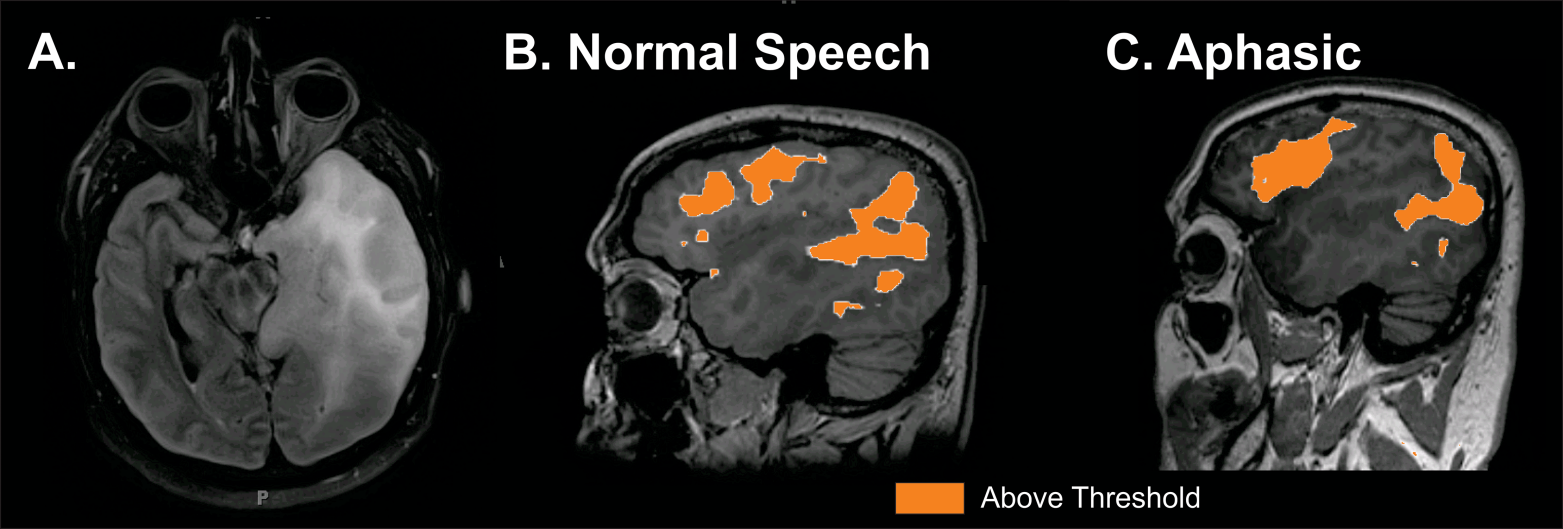
Resting state MRI mapping of speech networks in an aphasic patient. A. Forty year old patient with left temporal tumor. B. rs-fMRI mapping of speech when speech was intact. C. rs-fMRI mapping of speech when patient globally aphasic due to prolonged seizure. Imaging threshold for MLP rsfMRI was 97% probability.

Patient is a forty-year-old male who presented with headaches and speech difficulty. Further evaluation with MRI demonstrated an expansile left temporal tumor (Fig 5A). The patient was given steroids and his speech returned to normal. During this time a resting state MRI was obtained and speech networks were identified (Fig 5B). Prior to his scheduled surgery he had a prolonged seizure and was post-ictally globally aphasic for several days. During this time a repeat resting state MRI was obtained and speech networks were again identified despite his inability to speak (Fig 5C). These findings are significant because they provide an extreme example of being able to map function despite compromised cognitive function (i.e. mapping speech in the setting of an aphasia). Notably, the resting state network topographies were similar between imaging when speech was intact and when it was compromised.

#### 4. Mapping eloquent cortex in a sedated pediatric patient

**Fig 6:**
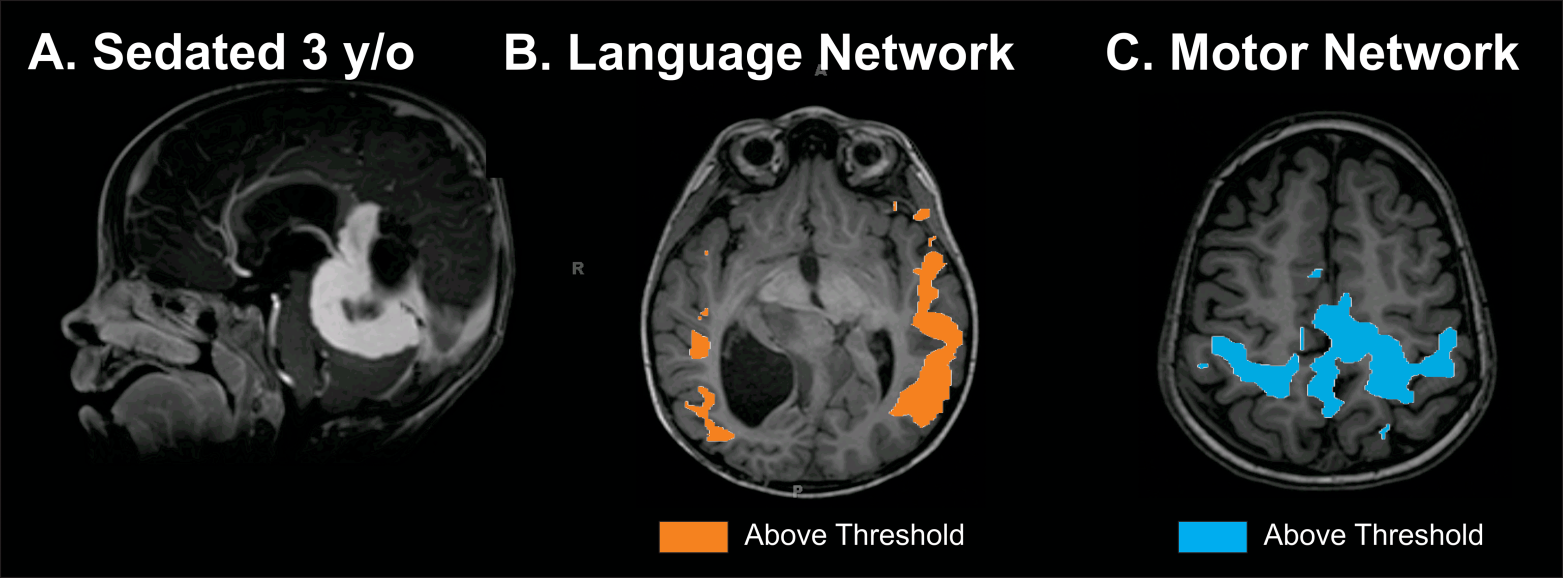
Resting state MRI mapping of eloquent cortex in a sedated pediatric patient. Three and half year old patient with brain stem tumor imaged while under propofol sedation. A. Tumor. B. rs-fMRI mapping of speech. C. rs-fMRI mapping of motor. Imaging threshold for MLP rsfMRI was 97% probability.

A three old boy with a prior history of pineal region fibroblastic spindle cell tumor that was previously resected who presented with behavioral changes and vomiting. For MR imaging he was sedated with propfol and sevoflourane. Imaging demonstrates a large tumor in the brain stem (Fig 6A), and motor and speech networks are clearly defined (Fig 6B and 6C). Consistent with prior studies in animals and humans, resting state networks are present despite alteration of consciousness with anesthetics[10, 27, 28]. It is also important to note that the patient is quite young and any form of task-based fMRI would not be possible.

## Discussion

The resting state fMRI analytics and IT work flow created at Washington University School of Medicine have converted a complex research-oriented brain mapping methodology into a practical and effective tool for preoperative assessment of functional regions in the brain. There are numerous advantages to resting state fMRI and its automated processing. Resting state fMRI does not require patient participation, numerous functional domains can be evaluated in a single imaging scan, and sedation does not alter the functional localization. The imaging pipeline associated with these analytics has enabled resting state fMRI to become a routine clinical tool. Additionally, we find in this institutional experience that the resting state fMRI performed much more reliably than classic task-based fMRI. Taken together, these findings support the important role that resting state fMRI can play in neurosurgical care.

### Advantages of a supervised machine learning for resting state MRI

Resting state fMRI analysis methodology historically was dominated by two complementary strategies: spatial Independent Components Analysis (sICA)[18], and seed-based correlation analysis (SCA)[7]. Both strategies assign resting state networks (RSNs) identities to brain voxels by exploiting the fact that spontaneous neural activity is correlated (coherent) within widely distributed regions of the brain. Both strategies yield highly reproducible results *at the group level in normal subjects*[22, 29]. sICA decomposes resting state fMRI data into a sum of components, each component corresponding to a spatial topography and a timecourse. Since sICA makes no *a priori* assumptions regarding the topography of RSNs, this method exemplifies unsupervised classification. The principal advantage of sICA is that it provides a direct means of separating artifact from blood oxygen level dependent (BOLD) signals of neural origin, although this separation typically requires observer expertise. However, the results obtained by sICA may vary substantially depending on processing parameters (e.g., number of requested components). Thus, sICA can be difficult to use in the investigation of targeted RSNs, especially in single subjects. In practice, the user must select the component of interest from the many typically returned by sICA, e.g., as in[30]. In contrast, seed-based correlation analysis (SCA) is computed by voxel-wise evaluation of the Pearson correlation between the timecourses and an a priori targeted region of interest (ROI) and all other voxels in the brain. The principal difficulty in using seed-based correlation mapping is exclusion of non-neural artifact, which typically is accomplished using regression techniques[14, 31, 32]. However, SCA many not be reliable when brain anatomy has been distorted by mass effects or RSNs have been rearranged to compensate for focal loss of function[33]. To address these shortcomings, we have previously described a perceptron-based method for mapping RSNs in individuals specifically designed for neurosurgical preoperative planning[9]. Perceptrons are supervised machine learning algorithms that can be trained to associate arbitrary input patterns with discrete output labels. We trained a multilayer perceptron (MLP) to associate seed-based correlation maps with particular RSNs. Running the trained MLP on correlation maps corresponding to all voxels in the brain generates voxel-wise RSN membership estimates. Several features of MLP-based RSN mapping make this technique highly suited to pre-surgical planning in tumor patients: (i) MLP outputs always represent the same entities, ordered in the same way; (ii) MLP-based RSN mapping, unlike unsupervised methods (e.g., sICA), is reliable in individuals; (iii) the MLP reliably assigns RSN membership in all voxels, even voxels not included in the training set; this property implies that the MLP accommodates focal anatomic distortions and rearrangements of RSN topography, which are expected in patients with brain tumors[9, 33]. Our current version of the MLP generates voxelwise membership estimates for the following seven RSNs: Default Mode network (DMN)[20, 21], Sensorimotor network (SMN)[7], Visual network (VIS)[18, 22], Language network (LAN)[18, 22], Dorsal and Ventral Attention network (DAN, VAN)[23, 24], Fronto-Parietal Control network (FPC). MLP-derived RSN estimation results can be displayed as seven continuous probability density maps or as one winner-take-all map. While the majority of maps used clinically in this institution experience were the motor, language, and visual networks; our mapping analytic enables the identification of all the functional domains, which may also potentially be clinically relevant for more subtle cognitive domains, such as attention and memory.

### The clinical advantages of using automated resting state fMRI mapping

Beyond the technical elegance of the MLP method efficiently localizing function despite brain lesions, the automated nature of the processing provides additional distinct clinical advantages. The resting state modality by itself enables functional imaging on patients who would typically be excluded from task-based fMRI due to an inability to cooperate with the various testing maneuvers required of task-based imaging. These patient populations include young children, cognitively impaired patients (based on either baseline disability or in the setting of a pathologic neurologic deficit), as well as in sedated patients. Additionally, we show examples that even patients who fail a task-based fMRI, the localization can still be accomplished with resting state. Another advantage created by the automation of the processing of resting-state fMRI data processed via a MLP algorithm is that it only requires 12 minutes of scanner time when completed in isolation. This is contrasted against the hour that is required for acquisition of task-based functional imaging (including the time to set up the equipment, train the patient and acquire the BOLD data). This superior functional imaging capability is seen in those patients who had both task and resting state imaging. The failure rate for resting state was significantly less than that of task-based fMRI, 13% vs. 38.5%, respectively. Additionally, traditional task-based fMRI requires specialized equipment for acquisition and processing of the data as well as personnel trained in acquiring the data and ensuring its quality prior to release of the patient from the radiology department. In addition to these resource utilization concerns, task-based studies can only be performed on a limited number of MR scanners, thus introducing limitations from a scheduling and departmental efficiency perspective as well. The radiology department must also commit to maintaining the equipment and providing trained staff to perform and process the exams. In contrast, the clinical resting-state fMRI protocol is available for acquisition on all 3-Tesla MR units at Washington University’s Barnes Jewish and St. Louis Children’s inpatient, outpatient, and research MR imaging departments and data can be acquired 24 hours per day, facilitating timely surgical intervention when necessary. Thus, the automated nature of the MLP algorithm reduces the need for committed technicians from the radiology department and increases the likelihood that this resting state MR technology can scale across institutions.

### The research advantages of using automated resting state fMRI mapping

Very often mapping techniques vary from institution to institution making it hard for insights from clinical experience to be rigorously compared and translated across practices. The standardized nature of the MLP mapping using resting state MRI ensures that functional localization is performed identically across all patients. Thus, the uniform mapping output can provide consistent localization information, such that various techniques (e.g. cortical stimulation, transcranial magnetic stimulation, and cortical physiologic changes) and various clinically relevant measurements (e.g. proximity of resection and neuropsychological/functional assessments) can all potentially be measured and compared across an identical mapping bench mark.

### Study limitations

There are several studies that are worth noting in this work. First, the study was retrospective in nature. Definitive conclusions of the manner in which resting state fMRI alters clinical practice and patient outcomes will require a prospective clinical study. Second, the current MLP-analytic and IT infrastructure was locally created and is not currently a commercial package that is widely available. Thus, wider scale implementation will require either a commercial product or dedicated institutional investment in creating a similar infrastructure. Third, while we have demonstrated successful execution of resting state fMRI in a clinical environment, this study does not have clinical outcomes data to assess whether the imaging altered or improved clinical care.

## Conclusion

Resting state fMRI is a useful presurgical planning tool that can be integrated into the clinical PACS system and the intraoperative neuronavigation system. It confers a distinct advantage in its ability to identify eloquent cortex in patients who would not be able to participate in a task-based study. Finally, when compared to classic task-based mapping, resting state MRI has a much lower failure rate.

## Supporting information

S1 File Imaging performance summary. Primary imaging outcomes for the patients studied are in this file.
(XLSX)

## Abbreviations

BOLD: Blood Oxygen Level Dependent
DWI: Diffusion-Weighted Imaging
MLP: Multilayer Perceptron
rs-fMRI: Resting-State Functional Magnetic Resonance Imaging
SWI: Susceptibility-Weighted Imaging (SWI)
fMRI: Functional Magnetic Resonance Imaging
